# Non-noxious skin stimulation activates the nucleus basalis of Meynert and promotes NGF secretion in the parietal cortex via nicotinic ACh receptors

**DOI:** 10.1007/s12576-014-0313-z

**Published:** 2014-05-07

**Authors:** Harumi Hotta, Nobuhiro Watanabe, Mathieu Piché, Sanae Hara, Takashi Yokawa, Sae Uchida

**Affiliations:** 1 Department of Autonomic Neuroscience, Tokyo Metropolitan Institute of Gerontology, 35-2 Sakaecho, Itabashi-ku, Tokyo, 173-0015 Japan; 2 Department of Chiropractic, Université du Québec à Trois-Rivières, Quebec, Canada; 3 BioView, Tokyo, Japan

**Keywords:** Physical stimulation, Nerve growth factor, Nicotinic receptor, Functional magnetic resonance imaging, Nucleus basalis of Meynert

## Abstract

The effects of non-noxious skin stimulation on nerve growth factor (NGF) secretion in the parietal cortex were examined in anesthetized rats. Innocuous skin stimulation was delivered to the left hindlimb with a soft-hair brush. Extracellular NGF in the right parietal cortex was collected by microdialysis methods using a protein-permeable probe and was measured using an enzyme-linked immune-sorbent assay. Brushing produced a significant increase in extracellular NGF levels. This NGF response was not observed in rats pretreated with a nicotinic ACh receptor (nAChR) antagonist mecamylamine. We further examined whether brushing could activate the basal forebrain nucleus (nucleus basalis of Meynert, NBM), which is the main source of cholinergic fibers in the cerebral cortex, by means of functional MRI. The blood oxygen level-dependent signal in the right NBM was significantly higher during brushing compared to baseline. The results suggest that non-noxious skin stimulation activates NBM and promotes NGF secretion in the parietal cortex via nAChRs.

## Introduction

Non-noxious mechanical skin stimulation produces not only somatosensory sensation but also has various effects on visceral functions independent of sensation in anesthetized animals and conscious humans [1–7]. Additionally, non-noxious skin stimulation may improve cognitive dysfunctions in patients with dementia [8–10], and motor functions in patients and animal models of cerebral infarction [11–15]. However, the mechanisms of these effects on the brain have not been clarified.

Nerve growth factor (NGF) is a neurotrophic factor that is produced and secreted by cerebral cortical neurons. Secreted NGF supports the survival and function of cholinergic basal forebrain neurons and cortical neurons essential for cognitive functions [16–19]. On the other hand, NGF secretion by cortical neurons is promoted by cholinergic inputs from the nucleus basalis of Meynert (NBM) [20]. Because of this important reciprocal influence between NGF secretion by cortical neurons and NBM cholinergic neurons, it is critical to determine what physiological stimulation promotes NGF secretion in the brain. However, there are only a few studies investigating NGF secretion in vivo due to the technical challenges of these methods. Therefore, in this study, we examined whether non-noxious mechanical cutaneous stimulation promotes NGF secretion in the parietal cortex in anesthetized rats using an in vivo microdialysis method with a protein permeable probe [21]. We hypothesized that non-noxious skin stimulation promotes NGF secretion in the cerebral cortex via the activation of the NBM.

We have shown that NGF secretion by cortical neurons is regulated by cholinergic inputs to the neocortex via nicotinic acetylcholine receptors (nAChRs) [20]. In anesthetized rats receiving unilateral electrical stimulation of the NBM for 100 min, NGF levels measured by enzyme-linked immune-sorbent assay (ELISA) in samples from a microdialysis probe in the parietal cortex increased ipsilaterally (up to 68 %) over prestimulus levels at 200–500 min after the stimulation ended. The NGF response was abolished by the nicotinic antagonist mecamylamine, but was unaffected by the muscarinic antagonist atropine [20]. Therefore, our second aim was to examine whether the effect of skin stimulation on NGF is produced by the activation of nAChRs.

Recently, many studies have examined spatial and temporal changes in neuronal activity in various brain regions using non-invasive brain imaging techniques such as functional magnetic resonance imaging (fMRI). Measurement of blood oxygen level-dependent (BOLD) signals by fMRI showed that the parietal cortex is activated by innocuous somatic afferent stimuli in humans and animals [22–27]. However, activation of the NBM by somatosensory stimulation has not been demonstrated by brain imaging. Therefore, we thirdly examined the effect of non-noxious skin stimulation on BOLD signal in the NBM and the parietal cortex using fMRI. Parts of the present results have been published in abstract form [28].

## Materials and methods

The experiments were performed on 18 male adult Wistar rats (3–6 months old, 300–410 g). All animal experiments were conducted with the approval of and in accordance with the Guidelines for Animal Experimentation prepared by the Animal Care and Use Committee of Tokyo Metropolitan Institute of Gerontology. Of the 18 rats, 13 were used for studies on NGF secretion and 5 were used for studies of fMRI. After the end of each experiment, rats were killed by injecting an overdose of pentobarbital.

Animals were anesthetized either with halothane (*n* = 12) or urethane (*n* = 6) as described in our previous studies [21, 29]. The trachea of each rat was cannulated and respiration was maintained by a mechanical ventilator. Rectal body temperature was maintained around 37.5 °C. Halothane anesthesia (1.5 % during the surgery, and fixed at 1.0 % after surgery) was used for most of the NGF experiment because this experiment requires a constant level of anesthesia for a long period of time. Urethane anesthesia (initially 1.1 g/kg, i.p.) was used for all the fMRI experiments and 1 of the NGF experiments. Additional doses of urethane were administered (0.2 g/kg, i.p.) to maintain an anesthetic level that included the absence of spontaneous motion, corneal and withdrawal reflexes, and spontaneous ventilation.

### Collection and measurement of extracellular NGF

All experimental conditions and techniques for collecting and measuring extracellular NGF in the parietal cortex using microdialysis and ELISA were similar to those described by Hotta et al. [21]. The animals were mounted in a stereotaxic instrument, and the right parietal bone and dura mater in an area of 3 mm in diameter at 3.5 mm lateral from the bregma was opened. A microdialysis probe covered with a protein permeable polysulfone membrane (NDP-I-4-03; EICOM, Kyoto, Japan) was inserted into the right parietal cortex with a lateral inclination of 30° to a depth of 3 mm from the cortical surface at 3.5 mm lateral from the bregma, and was perfused at a flow rate of 2 μl/min with 0.01 M phosphate buffered saline (PBS) using a pump (ESP-64; EICOM). The outlet of the probe was connected to a silicon tube, the tip of which was set at approximately 20 mm below the level of the cortical surface to help the outflow of the cortical perfusate by negative hydrostatic pressure. Cortical perfusate during a period of 40 min from the initiation of the perfusion, while the microdialysis probe and perfusion was stabilizing, was discarded, and we began collecting cortical perfusate every 100 min for 600 min. Each sample was stored at −20 °C until assayed. The NGF concentration was measured using an ELISA kit (Promega, USA) reported to detect mature NGF, but not pro-NGF [30]. We used samples twice-diluted with the sample buffer for the assay of NGF in cortical perfusate, and calculated the original concentration of NGF.

We have previously shown that NGF levels were stable throughout this experimental period in control condition without any stimuli [20, 21].

### MRI data acquisition and analysis

Animals were placed in a prone position and fixed into a handmade acrylic chamber. The animals’ head, chest, and legs were immobilized in the chamber, which was placed in the scanner. Images were acquired using a Varian volume coil (16 cm in diameter) with a handmade circular shaped receiver surface coil (2 cm in diameter) and a 4.7 T horizontal scanner interfaced with a Varian console (Unity INOVA 4.7 T). Functional MR data (BOLD response) were acquired using a single-shot gradient echo-planar imaging sequence [flip angle (FA) = 13°; repetition time (TR) = 5.008 s; echo time (TE) = 4 ms; field of view (FOV) = 3 × 3 cm; matrix = 32 × 32; number of slices = 5; slice thickness = 0.7 mm]. The 5 contiguous axial slices were collected in an interleaved manner and covered the hindpaw representation in the primary somatosensory cortex (from 2.75 mm posterior to 0.75 mm anterior to bregma). To examine brain activity induced by brushing stimulation, a block design of 29 min duration was used, including 14 stimulation blocks of 60 s, separated by rest periods of the same duration. High-resolution anatomical images were also acquired at the end of the experiment using a fast spin-echo sequence (TR = 2.5 s; TE = 13 ms; FOV = 3 × 3 cm; matrix = 256 × 128; number of slices = 5; slice thickness = 0.7 mm).

Images were analyzed using SPM 8 (Wellcome Trust Centre for Neuroimaging, London, UK; http://www.fil.ion.ucl.ac.uk/spm/). Preprocessing included realignment for movement correction and co-registration of functional images with anatomical images. Functional images were temporally filtered using a high-pass filter with a cutoff period of 128 s. Based on our hypothesis, a region of interest (ROI) analysis was performed on the BOLD signal in the left and right parietal cortex and left and right NBM. ROIs (circles of 1-mm in diameter) were placed in the parietal cortex (1.4 mm posterior, 2.5 mm lateral and 1.5 mm ventral to bregma) [31] and in the NBM (1.4 mm posterior, 3 mm lateral and 7.6 mm ventral to bregma). The location of the ROIs was determined based on a previous study that examined the effect of somatosensory stimulation applied to the hindlimb [29]. The ROI locations appear in Fig. 2.

### Stimulation of the skin

Innocuous stimulation using a soft-hair brush was applied to the posterior aspect of the left hindlimb covering the area from the buttock to the sole of the paw. Brushing was performed manually and was paced with an auditory cue. In our previous study [21], repetitive electrical stimulation of the NBM for 100 min produced an increase in extracellular NGF levels. Therefore, 1 Hz stimulation for 100 min was chosen for the NGF experiments. On the other hand, results of our blood flow study [29] suggested that 1 Hz brushing activates NBM with a longer latency (>2 min) than that of 3 Hz brushing (<10 s). Therefore, 3 Hz stimulation for 1 min was chosen for the fMRI experiments to repeat short-lasting stimulation for averaging and minimize the length of the experimental session. In the fMRI experiment, the animals’ lower thighs were fixed to the holder with adhesive tapes, with stimulation applied mainly to the paw.

### Drugs

In 7 NGF experiments, the nAChR antagonist mecamylamine (mecamylamine hydrochloride; Sigma, USA), which is blood–brain barrier (BBB) permeable, was used. Mecamylamine was dissolved in saline at a concentration of 20 mg/ml, and administered intravenously through a femoral vein as a single dose at 100 min before the onset of the brushing stimulation. Ficoll 70 (Pharmacia Biotech, Sweden) was injected (i.v.) to compensate hypotension following mecamylamine injection. In preliminary experiments, we confirmed that mecamylamine did not produce systematic changes in NGF levels in cortical perfusate throughout 600 min after injection.

### Statistical analysis

For statistical analysis, Prism 6 software (GraphPad Software, La Jolla, CA, USA) was used. Values are shown as mean ± SE. The changes in NGF and BOLD (separately for each ROI) evoked by brushing were assessed by a paired *t* test. The statistical significance level was set at *p* < 0.05.

## Results

### Changes in extracellular NGF levels in the cerebral cortex in response to brushing

**Fig. 1:**
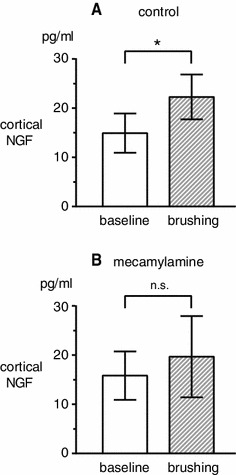
Changes in NGF concentrations in cortical perfusate in the right parietal cortex induced by brushing of the left hindlimb. NGF concentrations in cortical perfusate taken before (*white column*) and after (*hatched column*) brushing. Each *column and error bar* shows the mean ± SE. **a** Control rats without drugs (*n* = 6). **b** Rats pretreated with mecamylamine (*n* = 7). *p < 0.05; significant difference between the values before (baseline) and after brushing

Extracellular NGF levels in the parietal cortex were measured in a total of 6 rats in the control condition without cholinergic blocker. Five rats were anesthetized with halothane and 1 rat was anesthetized with urethane. No differences were found across the two different anesthetics on basal levels and brushing-induced changes in NGF. Therefore, all data were pooled across different anesthetics. In all rats examined, the NGF concentration did not change during brushing, but increased gradually after the end of the stimulation. The NGF concentration was maximum in samples taken at 200–400 min after the cessation of brushing. The NGF concentration before stimulation was 14.9 ± 4.0 pg/ml, and was increased significantly (*p* < 0.05) to 22.3 ± 4.6 pg/ml in samples taken at 300–400 min after the stimulation ended (Fig. 1a).

### Effects of a nAChR antagonist on the brushing-induced increase in extracellular NGF levels in the cerebral cortex

To define the role of cholinergic synapses in mediating brushing-induced increases in extracellular NGF, we examined whether a specific cholinergic receptor antagonist could reduce cortical responses to brushing. Mecamylamine, a nAChR antagonist, was used in 7 rats at 20 mg/kg (i.v.), which blocked the increase in NGF levels induced by NBM stimulation in our previous study [20]. Basal levels of extracellular NGF were 15.8 ± 4.9 pg/ml, which was similar to levels in control rats without the cholinergic receptor antagonist. However, the increase in extracellular NGF levels after brushing seen in control rats was not induced. The NGF concentration was 19.7 ± 8.3 pg/ml in samples taken at 300–400 min after the stimulation ended (Fig. 1b). The NGF levels did not show any significant change after brushing compared to pre-stimulus levels (*p* = 0.7).

### BOLD signal changes in the NBM in response to brushing

**Fig. 2:**
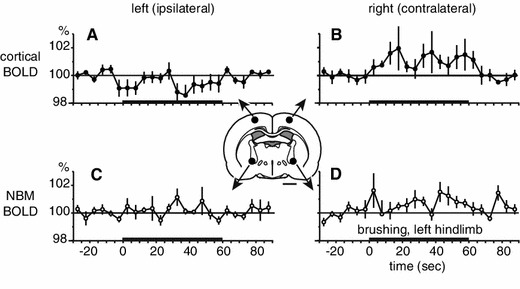
Changes in BOLD signals in the parietal cortex and the NBM induced by brushing of the left hindlimb. BOLD signals during the 2-min trial measured every 5 s in the parietal cortex (**a**, **b**) and the NBM (**c**, **d**), ipsilateral (**a**, **c**) and contralateral (**b**, **d**) to the stimulated hindlimb, expressed as a percentage of prestimulus control signal (*n* = 5). Averages of 30 s before the brushing are used as prestimulus control signal. The *thick horizontal lines* on the *abscissa* indicate the 1-min period of stimulation. BOLD signal was extracted from each ROI illustrated by *black circles in the inset* of a coronal slice at 1.4 mm posterior to bregma. *Scale bar below inset* 2 mm

The location of the measured ROIs is shown in the inset diagram of the rat brain in Fig. 2. BOLD signal changes in the parietal cortices and NBMs induced by 1-min non-noxious brushing at a frequency of 3 Hz were averaged in 5 rats. Non-noxious brushing was applied to the left hindlimb. During brushing, cortical BOLD signals increased in the right hemisphere on the contralateral side of the stimulated hindlimb (Fig. 2b). Changes in cortical BOLD signals occurred within 10 s from the onset of brushing and remained increased until the end of the stimulation. In contrast, BOLD signal in the ipsilateral parietal cortex did not increase (Fig. 2a).

**Fig. 3:**
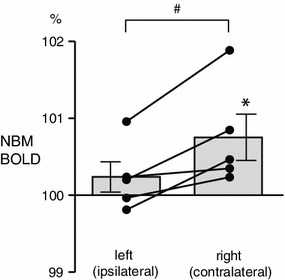
Changes in BOLD signals in the NBM during brushing of the left hindlimb. Mean values of BOLD during brushing are plotted as the percentage of prestimulus control value (averages of 30 s before stimulation). Each *column and error bar* shows the mean ± SE (*n* = 5). Each *point* indicates individual data. Each data obtained simultaneously is connected by a *line*. *p < 0.05; significantly different compared to the prestimulus control value (statistical analysis was performed using absolute values). ^#^ *p* < 0.05; significantly different between values

In the NBM contralateral to the stimulated hindlimb, the BOLD signal was increased during brushing (Fig. 2d). BOLD signals increase in the contralateral NBM was observed during the first 5 s and it remained increased throughout the stimulation period. The averaged BOLD signal during the whole brushing period was significantly higher than the prestimulus control signal (*p* < 0.05) (Fig. 3). The BOLD signal tended to increase in the ipsilateral NBM, but this effect did not reach statistical significance (*p* = 0.29) (Fig. 3). To further determine whether the NBM response was lateralized between left (ipsilateral side of stimulated hindlimb) and right (contralateral) hemispheres, the BOLD signal in the NBM of each hemisphere during brushing was compared (Fig. 3). Changes in the BOLD signal in the contralateral NBM were consistently and significantly larger compared to those of the ipsilateral NBM (*p* < 0.05).

## Discussion

Our results show that NGF secretion in the parietal cortex could be evoked by somatosensory stimulation. Our findings that (1) NGF increases are first detectable hours after brushing, and (2) the NGF response was reduced by a nAChR blocker are consistent with the NGF response induced by electrical stimulation of the NBM [20, 21]. Therefore, it is most likely that cholinergic inputs from the NBM contribute to the secretion of NGF by somatosensory stimulation. Accordingly, we also showed that hindlimb stimulation increased BOLD signal in the NBM.

Secreted NGF is essential for cholinergic neurons to maintain their properties [32, 33]. For example, i.c.v. injection of NGF reverses cholinergic neuron atrophy and improves the learning abilities of aged rats with impaired spatial memory [16]. In addition, cortical injection of NGF antibodies impairs acetylcholine release and disrupts the learning abilities in adult rats [18]. These studies together with our studies [20, 21] indicate that there is a bidirectional relationship between basal forebrain cholinergic neurons and the neurons in cortical areas where they project, through NGF. NGF prevents glucose deprivation-induced or glutamate-induced neuronal damage in cortical cell cultures [17, 34] at concentrations well within the estimated concentrations in the cortical extracellular fluid [20]. Therefore, the secretion of NGF induced by somatic sensory stimulation as shown in this study may be useful for maintaining the functions of cortical neurons and NBM cholinergic neurons essential for cognitive functions. The results of this study may explain why repeated somatosensory stimulation improves cerebral function in demented or post-stroke conditions [8–15]. Furthermore, non-noxious stimulation of the skin in daily life may contribute to maintain cognitive function by increasing NGF.

Non-noxious cutaneous stimulation may promote the secretion of various hormones and neurotransmitters, such as CRH [35], oxytocin [36], ACh [37], and dopamine [38], from neurons in the brain. However, our results are the first evidence indicating that somatosensory stimulation promotes the secretion of neurotrophic factors. Time delay between BOLD changes and NGF increase (this study) are similar to delay between electrical stimulation of the NBM and NGF increase [20], and can be explained by synthesis of NGF proteins via mRNA. Neeper et al. [39] showed that NGF mRNA levels increased in the hippocampus and caudal neocortex following 2–7 nights with running wheels. Thus, brushing of the hindlimb may also increase expression of the NGF in the parietal cortex.

In the present study, somatosensory-evoked BOLD signal changes were investigated by functional MRI. Innocuous brushing of the hindlimb induced an increase in the BOLD signal of the contralateral parietal cortex over the representation of the hindlimb, as expected from previous studies [22–26], and also of the contralateral NBM. This is the first study to show that somatosensory stimulus evoked BOLD signal changes in the NBM. The latency of the BOLD response in NBM was earlier than that in the parietal cortex. This result is consistent with the finding that blood flow increase in the parietal cortex induced by electrical stimulation of the NBM starts a few seconds after the onset of stimulation [40, 41]. Since an increase in BOLD signal usually correlates with an increase in neuronal activities [42, 43], the present result supports the above assumption that NBM neurons are activated by non-noxious cutaneous stimulation.

Changes in BOLD signal were significantly larger in the NBM in the contralateral compared with the ipsilateral side of the stimulated hindlimb. This result indicates that non-noxious skin stimulation activates the contralateral NBM predominantly. Accordingly, hindlimb brushing increases NGF secretion (this study) and ACh release measured on the contralateral parietal cortex [37]. Furthermore, a brushing-induced increase in regional blood flow evoked in the contralateral parietal cortex, over the representation of the stimulated hindlimb, was decreased in the hemisphere ipsilateral to muscimol inactivation of NBM [29]. In addition, histological studies have shown that fibers from the NBM project unilaterally to ipsilateral cerebral cortices [44, 45]. Furthermore, although NBM innervation of the somatosensory cortex is mostly cholinergic in the rat, and each cortical area receives cholinergic afferents from neurons widely distributed within the NBM, each NBM neuron projects to a restricted cortical area without significant collateralization to adjacent subdivisions [46]. However, ascending neural pathways from cutaneous mechanoreceptors to the contralateral NBM are unknown. Future studies are needed to clarify the specific neural pathway from the limbs to the contralateral NBM.

### Limitations

One limitation of this study is that the difference of stimulus frequency between the NGF studies (1 Hz for 100 min) and the fMRI studies (3 Hz for 1 min) may make it harder to link the BOLD signal results with the NGF data. However, in our previous experiments, brushing at 1 Hz continued for 15 min produced an increase of cortical blood flow equivalent to (or even larger than) that produced by brushing at 3 Hz for 3 min [29]. Therefore, it can be assumed that brushing at 1 Hz for 100 min would produce increases in BOLD signals equivalent to (or even larger than) that produced by brushing at 3 Hz for 1 min.

Another limitation is that we did not test the effect of a nAChR antagonist that does not permeate the blood–brain barrier, so that we cannot exclude the possibility that blocking transmission at autonomic ganglia that may influence sympathetic and parasympathetic tone are involved in the present NGF response. However, this possibility is less likely because autonomic nerve fibers innervate blood vessels on the cortical surface, but do not reach the cortical parenchyma. In addition, we did not examine the effect of mecamylamine on BOLD signal changes during brushing. However, we observed (data not shown) that brushing-induced increases in parietal cortical blood flow persisted after i.v. injection of mecamylamine (but were reduced by atropine). Therefore, the effect of mecamylamine on brushing-induced increases in NGF does not appear to be due to abolishing blood flow changes or transmission of somatosensory input from the hindlimb, but most likely to blocking nAChRs receiving ACh released from cholinergic fibers originating in the NBM.

## Conclusion

**Fig. 4:**
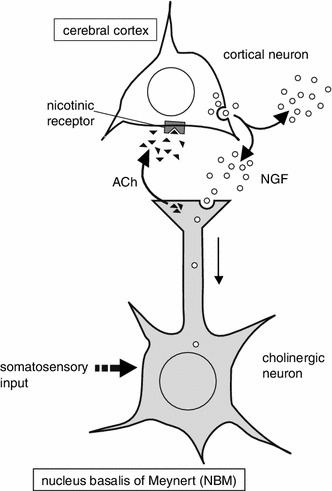
Schematic diagram showing the proposed mechanisms by which NGF secretion occurs in the cerebral cortex in response to non-noxious somatosensory stimulation. Non-noxious somatosensory input activates the NBM, and cholinergic inputs from there to the cortex promote NGF secretion from cortical neurons by activation of the nAChRs in the cerebral cortex

We propose that non-noxious mechanical stimulation applied to the skin activates the cholinergic system of the basal forebrain and promotes NGF secretion in the parietal cortex (Fig. 4). Secreted NGF may act to maintain neuronal function at the appropriate levels. Although direct evidence of neuronal excitation by means of electrophysiological technique is lacking, the present results on cortical NGF responses and BOLD changes in the NBM are consistent with and strongly suggest that non-noxious cutaneous stimulation activates the NBM neurons. The present animal fMRI study results may be advantageous for the translation towards human fMRI studies for screening appropriate stimulation that activates the NBM, leading to the maintenance of normal cognitive ability.

## Abbreviations

BOLD: Blood oxygen level-dependent
ELISA: Enzyme-linked immune-sorbent assay
fMRI: Functional magnetic resonance imaging
nAChRs: Nicotinic ACh receptors
NBM: The nucleus basalis of Meynert
NGF: Nerve growth factor
ROI: Region of interest
